# An Alternative Approach for Registration of High-Resolution Satellite Optical Imagery and ICESat Laser Altimetry Data

**DOI:** 10.3390/s16122008

**Published:** 2016-11-27

**Authors:** Shijie Liu, Yi Lv, Xiaohua Tong, Huan Xie, Jun Liu, Lei Chen

**Affiliations:** 1 College of Surveying and Geo-informatics, Tongji University, 1239 Siping Road, Shanghai 200092, China; 2009hello@tongji.edu.cn (Y.L.); huanxie@tongji.edu.cn (H.X.); 10junliurs@tongji.edu.cn (J.L.); 103495chen@tongji.edu.cn (L.C.); 2 Center for Spatial Information Science and Sustainable Development Applications, Tongji University, 1239 Siping Road, Shanghai 200092, China

**Keywords:** Antarctica, ASTER, ZY-3, ICESat, feature point, feature line, registration

## Abstract

Satellite optical images and altimetry data are two major data sources used in Antarctic research. The integration use of these two datasets is expected to provide more accurate and higher quality products, during which data registration is the first issue that needs to be solved. This paper presents an alternative approach for the registration of high-resolution satellite optical images and ICESat (Ice, Cloud, and land Elevation Satellite) laser altimetry data. Due to the sparse distribution characteristic of the ICESat laser point data, it is difficult and even impossible to find same-type conjugate features between ICESat data and satellite optical images. The method is implemented in a direct way to correct the point-to-line inconsistency in image space through 2D transformation between the projected terrain feature points and the corresponding 2D image lines, which is simpler than discrepancy correction in object space that requires stereo images for 3D model construction, and easier than the indirect way of image orientation correction via photogrammetric bundle adjustment. The correction parameters are further incorporated into imaging model through RPCs (Rational Polynomial Coefficients) generation/regeneration for the convenience of photogrammetric applications. The experimental results by using the ASTER (Advanced Spaceborne Thermal Emission and Reflection Radiometer) images and ZY-3 (Ziyuan-3 satellite) images for registration with ICESat data showed that sub-pixel level registration accuracies were achieved after registration, which have validated the feasibility and effectiveness of the presented approach.

## 1. Introduction

Remote sensing observation of the Antarctic ice sheets, compared to traditional in situ measurements, is an important and relatively efficient method to acquire the spatial information of the ice-sheet topography as well as its change, and provides essential information for the study of global climate change and sea level rise [1,2,3,4]. Satellite optical images and altimetry observations are the two main data sources used in Antarctic remote sensing. A number of radar altimetry satellites, such as Seasat (Sea Satellite), Geosat (Geodetic Satellite), ERS (European Remote Sensing Satellite), and Envisat (Environmental Satellite), had found applications in Antarctic research since its emergence. However, the radar altimetry data has accuracy challenge over rough topographic areas and small mountain glaciers or ice caps [5]. Relatively, laser altimetry can provide higher accuracy observation, e.g., the norminal accuracy of the ICESat (Ice, Cloud, and land Elevation Satellite) altimetry data is as high as 0.15 m [4]. However, the sparse distribution of the ICESat points leaves quite a large blank area without observations [6,7,8], resulting in uncertainty in mass balance estimation with spatial interpolation for the blank areas [8].

Optical remote sensing is another major data source used in the Antarctic research, which can provide high-resolution images with rich texture. The configuration of the along-track stereo cameras capable of recording near-simultaneous stereo images, can substantially improve the correlation between the stereo-pairs in extreme and changing environment [9]. Multi-temporal images over the same areas, on the other hand, allow interpretation of the ground changes [10]. To date, various satellite and aerial optical images have found applications in Antarctic ice surface evolution monitoring [5,11,12,13].

Satellite optical imagery has an advantage in texture description, and the accuracy of horizontal positioning is higher than that of height determination. With regard to radar and laser altimetry, however, the accuracy in height is higher than that in horizontal plane, which is complementary to optical images. Therefore, the integration of these two different types of datasets is expected to produce products with higher accuracy. However, due to the difference in platform and data acquisition mechanisms, there always exists geometric inconsistency between the two different types of datasets, which needs to be eliminated through registration for further integration processing [14,15].

There is lots of published research regarding the registration of optical images and point cloud data [15,16,17,18,19,20,21,22,23,24,25,26,27,28,29,30,31,32,33,34,35,36,37,38]. The methods can be classified into four categories [24], including feature based methods [19,20,26,32,33,34,35], mutual information based methods [23,36], frequency based methods [37,38,39], and salient point based methods [26,40]. As for feature based methods, the features can be categorized into corner points, straight lines, sensor invariant features, and building roofs, etc. Feature based methods may use one type or multiple types of features for registration [19,20,26,27,32]. Whatever feature types it may use, the registration is generally based on conjugate feature matching, such as point-to-point, line-to-line, or patch-to-patch—i.e., the same type features should be extractable in both datasets. As regards linear feature based registration, since lines are determined by points, the linear feature based method is implemented like point-to-line matching using coplanarity constraint. The registration can be solved in two ways. One is to use LiDAR (Light Detection and Ranging) features as control for establishing the datum in the photogrammetric bundle adjustment to correct the image orientation parameters [19,22,41], the other starts by 3D model construction from photogrammetric images and followed by a 3D similarity transformation in object space using the conjugate photogrammetric and LiDAR straight line features [19]. Even though the linear feature based registration is implemented like point-to-line matching, it requires conjugate linear features extractable in both the optical images and the LiDAR data. This is feasible for LiDAR point cloud which is dense enough for accurate location and extraction of the conjugate features. As regards the sparse ICESat point data, however, the extraction of conjugate features is a big challenge and even impossible. Therefore, the methods applicable for the registration of optical images and LiDAR data are probably infeasible for ICESat data.

The fusion of ASTER stereo images and ICESat altimetry data was investigated in [42] to produce a high accuracy digital elevation model (DEM) in East Antarctica, in which the geometric biases between the two datasets, however, was not carefully examined. A terrain-feature-based method was proposed in [43] to register the historical aerial images to ICESat data for measuring the elevation changes on Byrd Glacier, Antarctica. The registration was based on similarity transformation between the respective 3D Cartesian coordinate systems of the two datasets in object space, for which the control information—Including 3D terrain feature points and corresponding 3D terrain feature lines—Is extracted from the ICESat data and stereoscopically from the aerial images, respectively.

This paper presents an alternative simplified point-to-line feature based method in image space for the registration of satellite optical images and ICESat altimetry point data. It is based on direct discrepancy correction in image space through 2D transformation between the projected terrain feature points and the corresponding 2D image feature lines. The method can work for mono images and requires no conjugate points, which facilitates the registration of sparse points and the optical images. Following the detailed methodology of the approach, comprehensive experiments using the ASTER images and ZY-3 images for registration with ICESat altimetry data were conducted and the registration results are demonstrated and discussed, leading to the conclusion in the final section.

## 2. Point-to-Line Registration of Satellite Optical Images and ICESat Altimetry Data

**Figure 1:**
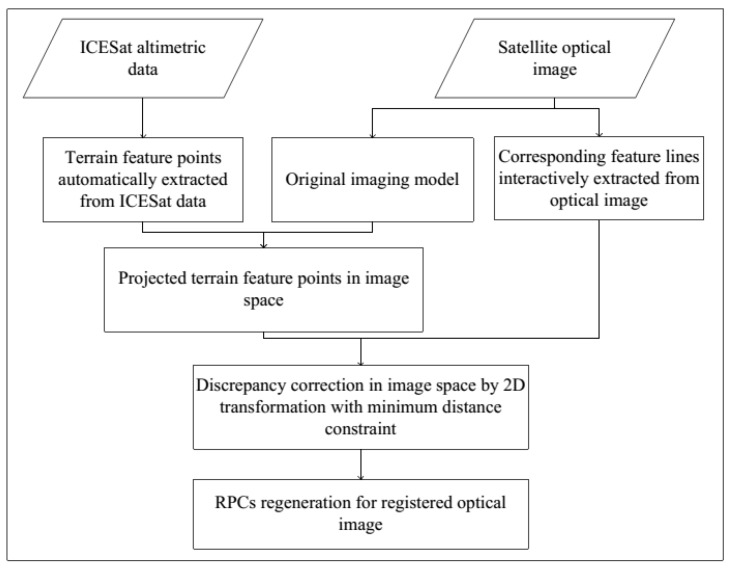
Framework of the methodology.

Figure 1 illustrates the framework of the methodology for the registration of satellite optical images and ICESat altimetry data. The method consists of three main steps: (1) extraction of the matching primitives including 3D feature points automatically extracted from ICESat data and corresponding 2D feature lines interactively extracted from optical images; (2) projection of the 3D feature points to image space and transformation between the projected points and the corresponding feature lines based on point-to-line distance minimization constraint; and (3) regeneration of the satellite imaging model by calculating the bias-compensated RPCs (Rational Polynomial Coefficients) incorporating the transformation parameters determined in step 2.

### 2.1. Extraction of Matching Primitives

**Figure 2:**
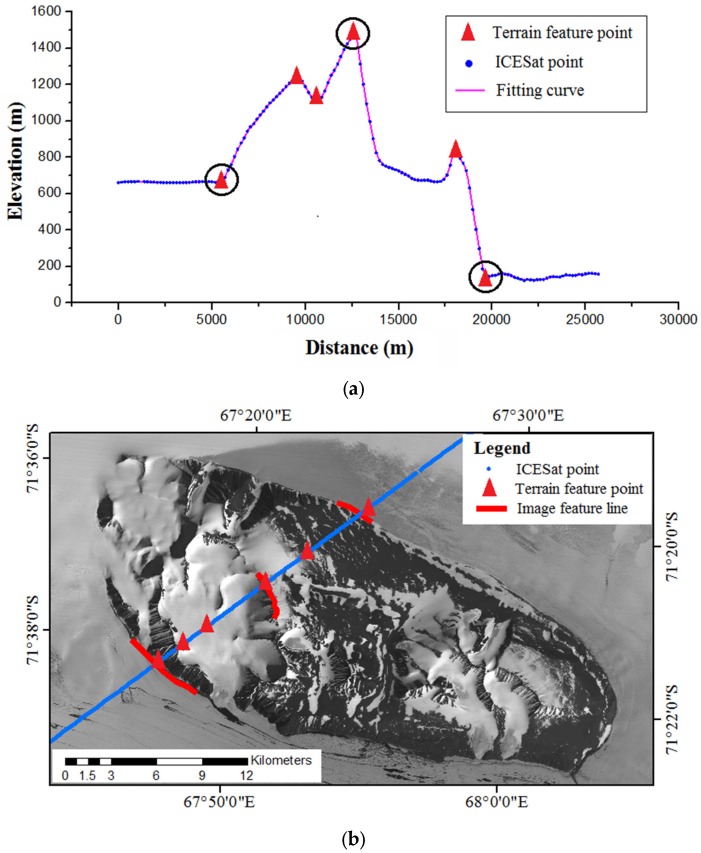
(**a**) Terrain feature points automatically extracted from Ice, Cloud, and land Elevation Satellite (ICESat) data, the circled points indicate that corresponding feature lines are extractable in optical image; (**b**) Corresponding terrain feature lines interactively derived from optical image.

Because the ICESat laser points are sparse, it is difficult and even impossible to identify the individual conjugate points from both the ICESat data and the optical images. However, both datasets contain terrain information, thus it is possible to obtain terrain features from both of them [43]. Terrain feature points such as abrupt slope changes, can be extracted from the elevation profiles along ICESat tracks. These feature points often correspond to ridge crests or foot boundary points, as illustrated in Figure 2a. The terrain feature points are calculated through curve fitting using the adjacent several laser points, thus they are more robust and accurate than direct laser observation points [43]. This is implemented automatically by setting a threshold value of slope change, and all the terrain feature points with a slope change value larger than the threshold will be automatically extracted. Then these terrain feature points are projected into the image space providing initial locations for guidance of corresponding image feature lines extraction. The corresponding image feature lines are extracted interactively with the aid of edge detection algorithms such as the Canny algorithm [44]. Due to the difference of radiation intensity caused by terrain fluctuation and surface material variation, some feature lines may be not distinct and of high uncertainty. These feature lines will be abandoned and only those distinct and of high certainty will be kept, as illustrated in Figure 2b. These feature lines correspond to ridge crest lines or foot boundary lines. The length of the extracted feature lines should be long enough that after transformation, the transformed feature points will coincide with the corresponding feature lines within the expected error limit.

It should be noted that the ground features, especially those ice-rock boundary lines, may have moved due to the glacier change between the ICESAT measurements and the image acquisitions. In order to minimize the registration error introduced by possible glacier change between the acquisition times of different datasets, we chose the ICESat observations which were obtained close to the time or in the same season of the satellite image acquisition for the extraction of conjugate terrain features. In addition, a good distribution of the terrain features in the full image extent contributes to a robust and higher registration accuracy.

### 2.2. Discrepancy Correction and Data Registration

The registration of ICESat laser points and optical images is based on the constraint that the projected terrain feature points extracted from ICESat data should coincide with the corresponding 2D feature lines measured from optical images. The 3D terrain feature points are first projected into image space via the vendor-provided imaging model—e.g., the RPCs provided along with the images. Due to the platform diversity and the errors in satellite orbit and attitude determination as well as inaccuracy of sensor calibration, there exist discrepancies between the projected terrain feature points and the measured feature lines in image space.

Generally, there are two ways to eliminate the geometric discrepancies. One is to correct the resulting discrepancies directly in image space or object space, which can be referred to as the direct method. The other is to correct the imaging model parameters through photogrammetric bundle adjustment to achieve geometric consistency between the two types of datasets, which can be referred to as the indirect method. In the latter method, the parameters and observations which cause the discrepancies are adjusted, including satellite attitude and orbit observations (referred to as exterior orientation) and sensor calibration parameters (referred to as interior orientation). The indirect method requires an initial rigorous sensor model and is of high computational complexity. It is impracticable if only the RPCs are available. The direct method, on the other hand, is simpler for comprehension and easier for computation, and is applicable for both a rigorous sensor model and RPC model. According to the space where the discrepancies considered and corrected, there are two schemes for the direct method. One is to correct the discrepancies in image space and the other is in object space, the latter one was adopted in the research in [43]. The object-space-based scheme requires stereo images to retrieve the 3D information and may fail to work with mono-view images, while it is of no problem for the image-space-based scheme. Discrepancy correction in image space performs much better than that in object space in the case of multisource image data [45].

The discrepancies between the projected terrain feature points and the measured feature lines in image space can be modeled and compensated by polynomial transformation with constraint of minimizing the distance between points and corresponding lines. Let us say that $\left(x_{i},y_{i}\right)$ are the image coordinates of the *i*th projected terrain feature point, $\left(x_{ci},y_{ci}\right)$ are the image coordinates after transformation, and $a_{i}x+b_{i}y+c_{i}=0$ is the corresponding feature line measured in image space. We have

(1) $$\left(\begin{array}{c} x_{ci}\\ y_{ci} \end{array}\right)=\left(\begin{array}{c} k_{x0}\\ k_{y0} \end{array}\right)+\left(\begin{array}{cc} k_{x1} & k_{x2}\\ k_{y1} & k_{y2} \end{array}\right)\left(\begin{array}{c} x_{i}\\ y_{i} \end{array}\right)$$

where $k_{xj}$ and $k_{yj}$ (*j* = 0,1,2) are the polynomial transformation parameters. According to the selection of the parameters, we have different transformation models as follows.

(a) $\left(\begin{array}{c} x_{ci}\\ y_{ci} \end{array}\right)=\left(\begin{array}{c} k_{x0}\\ k_{y0} \end{array}\right)+\left(\begin{array}{c} x_{i}\\ y_{i} \end{array}\right)$, translation model with two parameters.
(b) $\left(\begin{array}{c} x_{ci}\\ y_{ci} \end{array}\right)=\left(\begin{array}{c} k_{x0}\\ k_{y0} \end{array}\right)+\left(\begin{array}{cc} k_{x1} & 0\\ 0 & k_{y1} \end{array}\right)\left(\begin{array}{c} x_{i}\\ y_{i} \end{array}\right)$, translation and scale model with four parameters.
(c) $\left(\begin{array}{c} x_{ci}\\ y_{ci} \end{array}\right)=\left(\begin{array}{c} k_{x0}\\ k_{y0} \end{array}\right)+\left(\begin{array}{cc} k_{1} & k_{2}\\ -k_{2} & k_{1} \end{array}\right)\left(\begin{array}{c} x_{i}\\ y_{i} \end{array}\right)$, similarity transformation model with four parameters.
(d) $\left(\begin{array}{c} x_{ci}\\ y_{ci} \end{array}\right)=\left(\begin{array}{c} k_{x0}\\ k_{y0} \end{array}\right)+\left(\begin{array}{cc} k_{x1} & k_{x2}\\ k_{y1} & k_{y2} \end{array}\right)\left(\begin{array}{c} x_{i}\\ y_{i} \end{array}\right)$, affine model with six parameters.

Generally, higher order polynomials are not necessary and the affine model is efficient enough to model and compensate the discrepancies [46,47].

For each pair of matched features, we have distance observation equation:

(2) $$v_{i}=\frac{a_{i}x_{ci}+b_{i}y_{ci}+c_{i}}{\sqrt{a_{i}{}^{2}+b_{i}{}^{2}}}$$

In Equation (2), $a_{i},b_{i},c_{i}$ are the known coefficients of the extracted image feature lines. Combine Equations (1) and (2) to replace $\left(x_{ci},y_{ci}\right)$ with image coordinates $\left(x_{i},y_{i}\right)$ of projected terrain feature point and the unknown transformation parameters $k_{xj}$ and $k_{yj}$. The transformation parameters can be solved using the least-square minimization constraint $\min(\overset{n}{\underset{i=1}{\sum }}v_{i}^{2})$, and n should be more than the number of model parameters for a steady and accurate solution.

For n pairs of matched features, the distance observation equations can be written in the form of:

(3) V=A⋅X−L

where X represents the vector of the to-be-solved transformation parameters k’s, A is the corresponding coefficient matrix, L is the vector of the remaining invariants, and V represents the vector of residuals. If we have *n* points, and k’s are the affine model parameters, the constructions of A, X and L are as follow:

$$A=\left(\begin{array}{cccccc} \frac{a_{1}}{\sqrt{a_{1}^{2}+b_{1}^{2}}} & \frac{a_{1}x_{1}}{\sqrt{a_{1}^{2}+b_{1}^{2}}} & \frac{a_{1}y_{1}}{\sqrt{a_{1}^{2}+b_{1}^{2}}} & \frac{b_{1}}{\sqrt{a_{1}^{2}+b_{1}^{2}}} & \frac{b_{1}x_{1}}{\sqrt{a_{1}^{2}+b_{1}^{2}}} & \frac{b_{1}y_{1}}{\sqrt{a_{1}^{2}+b_{1}^{2}}}\\ \frac{a_{2}}{\sqrt{a_{2}^{2}+b_{2}^{2}}} & \frac{a_{2}x_{2}}{\sqrt{a_{2}^{2}+b_{2}^{2}}} & \frac{a_{2}y_{2}}{\sqrt{a_{2}^{2}+b_{2}^{2}}} & \frac{b_{2}}{\sqrt{a_{2}^{2}+b_{2}^{2}}} & \frac{b_{2}x_{2}}{\sqrt{a_{2}^{2}+b_{2}^{2}}} & \frac{b_{2}y_{2}}{\sqrt{a_{2}^{2}+b_{2}^{2}}}\\ \vdots & \vdots & \vdots & \vdots & \vdots & \vdots \\ \frac{a_{n}}{\sqrt{a_{n}^{2}+b_{n}^{2}}} & \frac{a_{n}x_{n}}{\sqrt{a_{n}^{2}+b_{n}^{2}}} & \frac{a_{n}y_{n}}{\sqrt{a_{n}^{2}+b_{n}^{2}}} & \frac{b_{n}}{\sqrt{a_{n}^{2}+b_{n}^{2}}} & \frac{b_{n}x_{n}}{\sqrt{a_{n}^{2}+b_{n}^{2}}} & \frac{b_{n}y_{n}}{\sqrt{a_{n}^{2}+b_{n}^{2}}} \end{array}\right)$$

$$X={\left(\begin{array}{cccccc} k_{x0} & k_{x1} & k_{x2} & k_{y0} & k_{y1} & k_{y2} \end{array}\right)}^{T}$$

$$L={\left(\begin{array}{cccc} \frac{-c_{1}}{\sqrt{a_{1}^{2}+b_{1}^{2}}} & \frac{-c_{2}}{\sqrt{a_{2}^{2}+b_{2}^{2}}} & \cdots & \frac{-c_{n}}{\sqrt{a_{n}^{2}+b_{n}^{2}}} \end{array}\right)}^{T}$$

The least-square solution of Equation (3) is:

(4) $$X={\left(A^{T}A\right)}^{-1}\cdot \left(A^{T}L\right)$$

In order to remove the possible outliers for a robust estimation, 3-sigma (3 σ) criterion is applied and those features with residuals more than 3 σ ($\sigma =\sqrt{\frac{V^{T}V}{n-t}}$, *t* is the number of transformation parameters) will be abandoned [48].

With application of the transformation model, the inconsistency between the two datasets can be minimized within an expected error to achieve data registration.

### 2.3. Imaging Model Regeneration for Optical Images

With the discrepancies being compensated by direct correction in image space, registration of the two datasets is achieved. However, the correction model always has to be provided along with the image, which is not convenient for applications. In order to incorporate the correction parameters, the original imaging model has to be updated.

Compared with the rigorous sensor models, the RFM (Rational Function Model, also called RPC model) is more popular and supported by most satellite image vendors and photogrammetric software. Therefore, bias correction can be incorporated into the photogrammetric model by generation/regeneration of a new set of bias-free RPCs. There a lot of studies have been published on RFM-based geometric performance evaluation and bias correction for various high-resolution satellite images [49,50,51,52,53]. The generation of a new set of bias-free RPCs includes the following steps [54].

(1) Establishment of a three-dimensional (3D) lattice in ground space. The lattice contains *m* × *n* × *k* (*m* and *n* represent the number of rows and columns in horizontal plane and *k* is the number of elevation layers) virtual object points. The dimension of the lattice should cover the range of the 3D terrain surface within the full extent of the image. To achieve a robust and accurate solution, the number of elevation layers should be greater than three, and the total number of object points should be adequate enough (e.g., 100) considering 78 to-be-solved RPC parameters.
(2) Determination of the corresponding image points with the bias being corrected. The virtual object points are projected into image space by using the original imaging model and are corrected by use of the image offset model parameters obtained in Section 2.2 to get the corrected image points.
(3) RFM fitting. The new bias-free RPC parameters are then solved using the corresponding image and object grid points by least-square adjustment, during which the original RPCs (if available) serve as initial values.

## 3. Experiments and Analysis

### 3.1. Data Used

**Figure 3:**
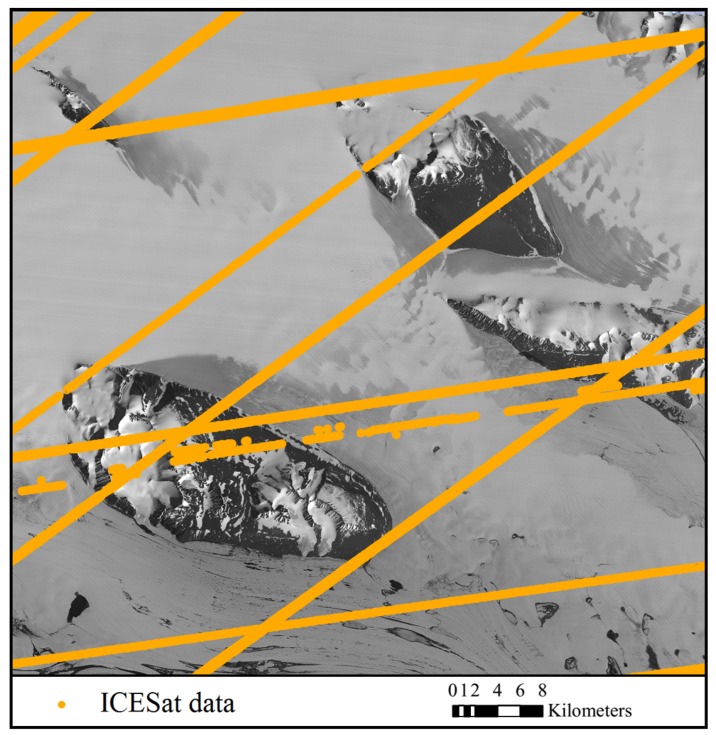
The nadir Advanced Spaceborne Thermal Emission and Reflection Radiometer (ASTER) image (recorded in January 2005) and distribution of the ICESat data (acquired between 2003 and 2009).

**Figure 4:**
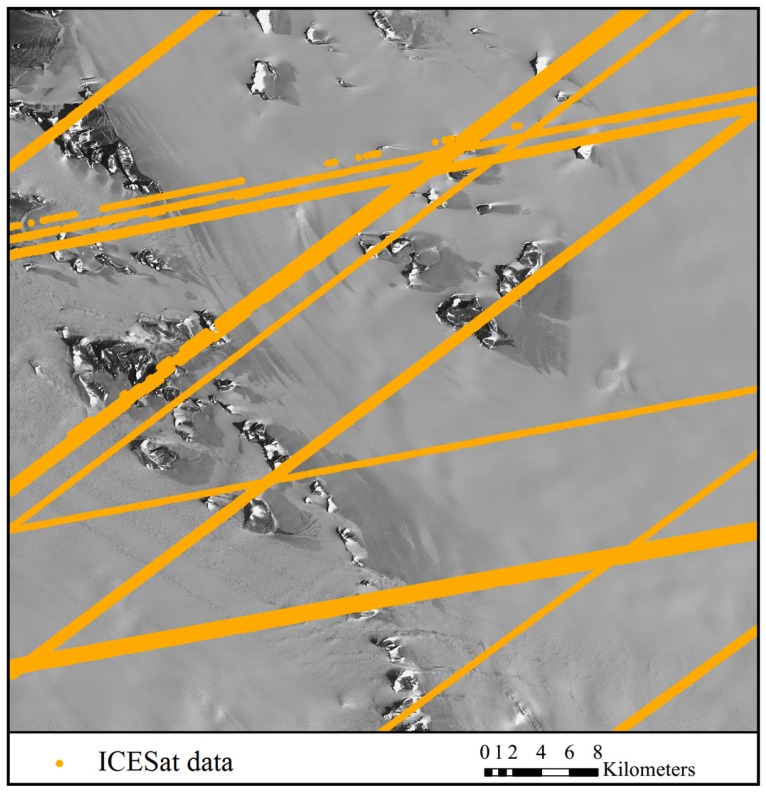
The nadir ZY-3 image (recorded in March 2014) and distribution of the ICESat data (acquired between 2003 and 2009).

The satellite optical images used for experiments include ASTER VNIR (Visible and Near Infrared Radiometer) images and Chinese ZY-3 satellite images. The ASTER images were acquired from the Earth Resources Observation and Science Center (EROS) of USGS (United States Geological Survey, Reston, VA, USA) (http://glovis.usgs.gov/). The images were level 1A products in HDF (Hierarchical Data Format) format, with nominal resolution of 15 m and geolocation accuracy of 72 m [55]. They were recorded in January 2005, one in nadir view and the other in backward view. Along with the ASTER image data, the spacecraft ancillary data and instrument supplementary data were provided, from which an RPC model can be generated by use of an ENVI software (Ver. 5.1) to facilitate further bias correction and regeneration of a new set of bias-free RPCs. The ZY-3 satellite, which was launched in 2012, is the first civilian high-resolution stereo mapping satellite of China. The ZY-3 images were provided by Satellite Surveying and Mapping Application Center (SASMAC). The ZY-3 images used in the experiments were sensor-corrected products in TIFF (Tag Image File Format) format, with nominal horizontal geolocation accuracy of 50 m. The images were acquired in March 2014, one in nadir view and the other in forward view, with resolutions of 2.1 m and 3.5 m, respectively. RPCs are provided along with the ZY-3 images for orientation. Both of the study areas are situated in east Antarctica near Lambert glacier as shown in Figure 3 and Figure 4. There are mountains and rocks in the image areas which provide terrain features for the registration of the optical images and the ICESat data.

The ICESat data was obtained from the National Snow and Ice Data Center (NSIDC). It was acquired between 2003 and 2009 by the onboard Geoscience Laser Altimeter System (GLAS). The altimeter samples 50–70 m diameter footprints every ~172 m along the track, with an elevation accuracy of about 0.15 m, and a pointing knowledge accuracy of ~2 arcsec, equivalent to 6 m horizontal geolocation error [56,57]. Considering that the vertical and geolocation accuracy of the ICESat data is higher than that of the satellite images, the ICESat data is treated as reference, and the satellite images is going to be co-registered to it. The satellite images overlayed with the ICESat data are shown in Figure 3 and Figure 4, which reveal a quite sparse distribution of the ICESat data, with large blank areas lacking altimetry observation between ICESat tracks.

### 3.2. Matching Primitives Extraction

**Figure 5:**
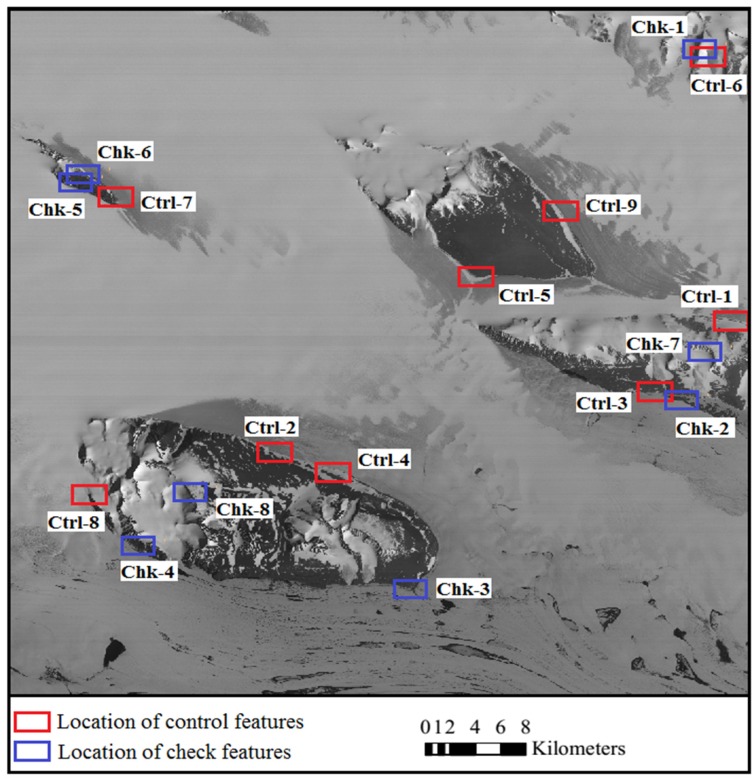
Distribution of the control features and the check features for the ASTER images.

**Figure 6:**
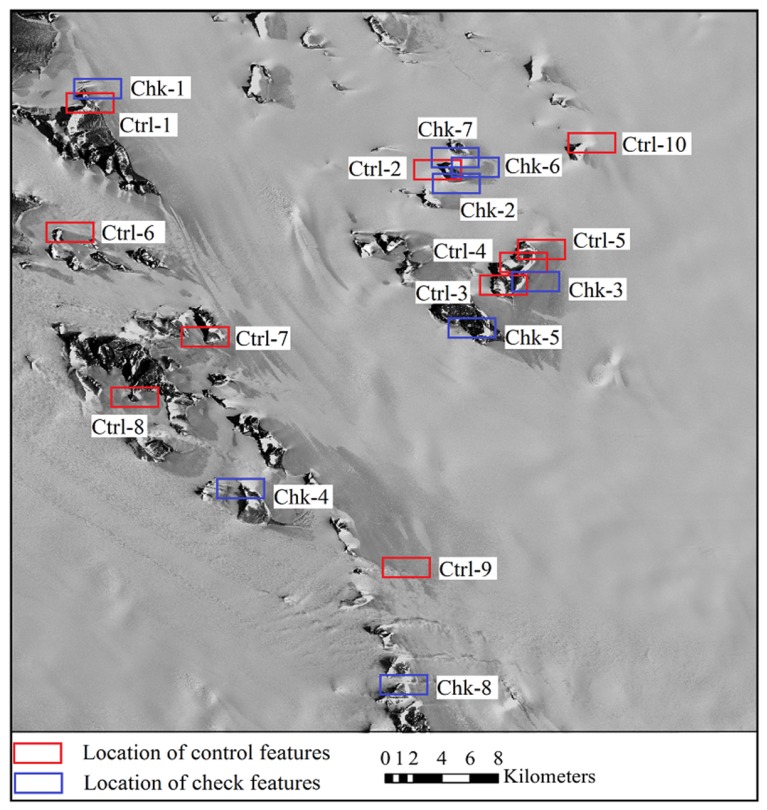
Distribution of the control features and the check features for the ZY-3 images.

The matching primitives, including the terrain feature points and the corresponding feature lines, were extracted from the ICESat altimetry data and the optical images respectively following the principle described in Section 2.1. As the ASTER images were acquired in January 2005, and the ZY-3 images were acquired in March 2014, the terrain feature points used as control for registration were mainly extracted from the ICESat laser points which were acquired in the same season close to January for ASTER and March for ZY-3. In the experiments, for the registration of ASTER images and ICESat data, nine pairs of terrain feature points and corresponding feature lines were extracted as control for each image, and another eight pairs were extracted as check for independent accuracy evaluation for each image. For the registration of ZY-3 images and ICESat data, the number of control features and check features are 10 and 8, respectively. The two images in each stereo pair share the same ground points for both the control features and check features. Figure 5 and Figure 6 show the distribution of the control features and the check features for the ASTER images and ZY-3 images, respectively.

### 3.3. Registration Results

**Figure 7:**
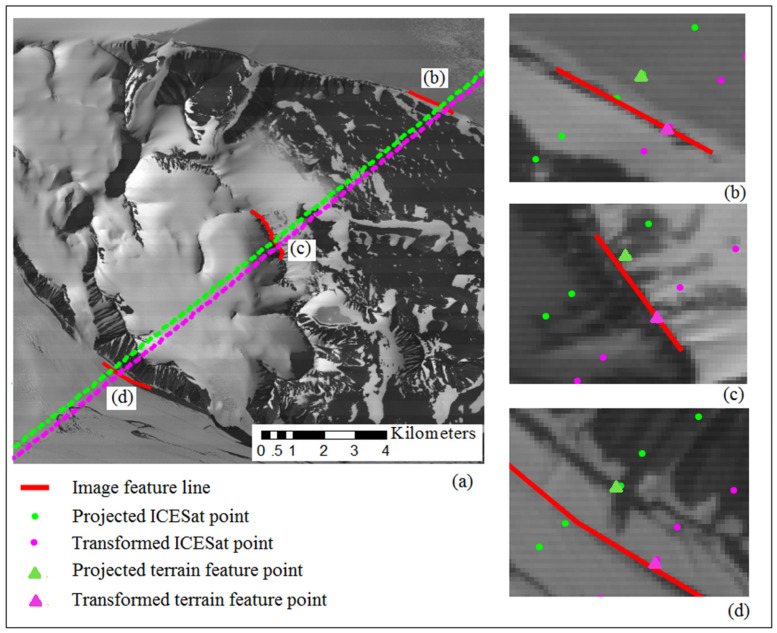
Discrepancies between the projected ICESat terrain feature points and the corresponding feature lines in the ASTER image before and after registration.

By using the extracted control features, the transformation model parameters can be determined for each image by least squares adjustment as introduced in Section 2.2. Different scenarios of parameterization were tested to compare the effectiveness of the four transformation models indicated in Section 2.2 and the results are listed in Table 1, from which we can see that before registration, the average discrepancies are more than four pixels between the ASTER images and the ICESat data. Among the four transformation models, the affine model produced the highest accuracy to sub pixel level after registration. The ZY-3 images showed relatively smaller inconsistency with the ICESat data at around 1–2 pixels before registration, and it was further improved to sub-pixel level after registration by using the similarity or affine transformation model. Figure 7 illustrates an example showing the geometric relation between an ICESat track and the corresponding ASTER image feature lines before and after registration, which reveals that a higher consistency between the ICESat terrain feature points and the corresponding image feature lines is achieved after registration.

**Figure 8:**
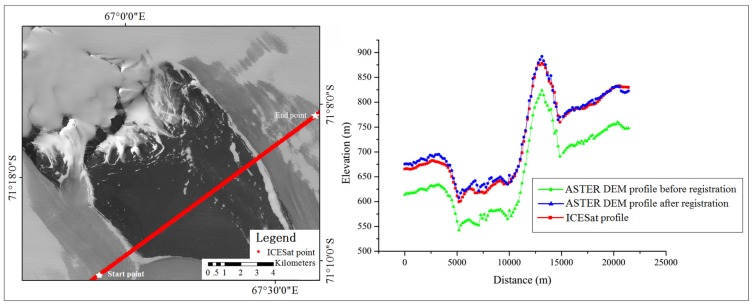
Profile comparison along an ICESat track between the ICESat data and the derived Digital Elevation Models (DEMs) from the ASTER images before and after registration.

**Figure 9:**
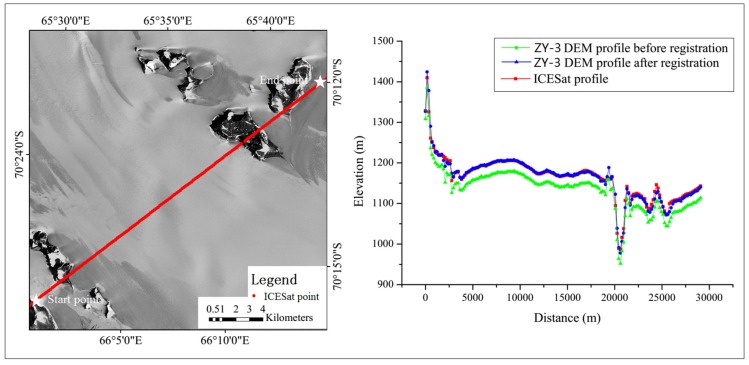
Profile comparison along an ICESat track between the ICESat data and the derived DEMs from the ZY-3 images before and after registration.

As discussed in Section 2.3, the transformation model needs to be fused into the imaging model through RPCs generation/regeneration for the convenience of applications. Based on the original imaging models and the derived affine transformation model, RPCs were generated for each optical image. In order to validate the accuracy improvement of the regenerated RPCs in photogrammetric applications, two DEMs (Digital Elevation Models) were generated from the stereo images by aid of an ERDAS (Earth Resource Development Assessment System) software, one using the original imaging model and the other using the regenerated bias-free RPCs. The two DEMs were then compared with the ICESat observations for consistency assessment before and after registration. Figure 8 and Figure 9 illustrate two elevation profiles along the ICESat tracks for the ASTER image and ZY-3 image, respectively, from which we can see that significant offset biases exist between the ICESat elevations and the photogrammetric elevations before registration, and a higher consistency was achieved between the ICESat data and the elevations derived from the optical images using the regenerated bias-free RPCs after registration. The mean and standard deviation of the elevation discrepancies before and after registration are listed in Table 2. From Table 2, we can see that the mean elevation discrepancies were significantly reduced from −61.4 m to 5.9 m for ASTER and from −28.3 m to −1.4 m for ZY-3, both better than the corresponding image resolutions of 15 m and 2.1 m/3.5 m, respectively. As regards the standard deviation, the improvement for ASTER was greater than for ZY-3, revealing that the geometric discrepancy between the ICESat and ASTER data is more complex than that between the ICESat and ZY-3 data before registration and more parameters are needed for the correction of ASTER data, which is in agreement with the results obtained in Table 1.

## 4. Discussion

Due to the difference in platform and systematic errors inherent in sensor calibration and orientation observations, geometric inconsistency exists between two different types of datasets, even between datasets obtained at different times from the same sensor, for which data registration is an essential procedure to eliminate the geometric inconsistency for integration use. Generally, translation could compensate a majority part of the inconsistency, which is corresponding to the discrepancies induced by constant sensor calibration errors and constant orbit and attitude measurement errors. However, there may also exist additional discrepancies after translation compensation, which could be attributed to the drift and/or higher errors with orbit and attitude measurements [45,49]. Therefore, to compensate the additional discrepancies, more parameters are needed for correction, such as translation and scales, similarity transformation, and affine transformation, as tested in the experiments in Section 3.3. Theoretically, the more parameters used in transformation, the smaller residuals it will produce, and the larger number of control features it requires. However, the transformation selected should be as close as possible to the physical model in order to avoid an over-fitting problem. Generally, if only the residuals meet the accuracy expectation, the number of parameters should be as smaller as possible to avoid over fitting. In our experiments, the expected registration accuracy is sub-pixel level. Therefore, for the registration of ASTER images and ICESat data, the affine transformation model is required to achieve the expected sub-pixel accuracy. For the registration of ZY-3 images and ICESat data, however, the similarity transformation model is adequate enough, although the accuracy could be further improved by using the affine model.

With the geometric inconsistency being corrected by the transformation model in image space, the registration of the two datasets is achieved. However, the use of additional transformation model is not convenient in application such as DEM generation. Therefore, the transformation parameters are incorporated into the imaging model through RPCs generation/regeneration. The results of DEM discrepancy analysis along ICESat tracks conducted in Section 3.3 validated the effectiveness of the updated imaging models.

A similar terrain-feature-based method was presented to register the historical aerial images to ICESat data in [43]. It was based on 3D similarity transformation with point-to-line distance minimization constraint in object space to transform the local photogrammetric system to ICESat reference system, requiring that the 3D model of terrain features be first generated stereoscopically from the aerial images. In the experiment, after a rigid body transformation adjustment with a total of 15 characteristic lines and points, the geometric inconsistency between the ICESat data and the 3D model generated from the aerial images were decreased from the original more than 100 m down to several meters. The method in [43] shared similar concept with the method proposed in [19] for the registration of images and lidar data based on linear features. They both start by manipulating the photogrammetric images to produce a 3D model including a set of linear features, and followed by establishment of the transformation between the two coordinate systems based on the control features. The advantage is that they can be applied for the co-registration of multiple three-dimensional datasets regardless of their origin. However, the disadvantage also lies in the requirement of three-dimension of both datasets being considered for co-registration—i.e., they require stereo images for 3D model construction and could not work for mono images. Moreover, the extraction of corresponding linear features from stereo images for 3D construction may encounter difficulty due to occlusions since the linear features are along object space discontinuities [19]. The alternative simplified point-to-line feature matching method presented in this paper is based on direct discrepancy correction in image space for the registration of satellite optical images and ICESat data, which is simpler and easier for implementation, and it can work for individual images, no matter stereo or mono. Meanwhile, discrepancy correction in image space also allows for investigation of the satellite stability—e.g., the affine correction model indicates the potential existence of gyro drift error and radial ephemeris error [46,49]. Because the methods discussed above were also not fully automatic, the implementation of these methods is therefore not conducted for comparison in the experiments since the major differences lie in conception and its limitation rather than the accuracies achievable.

## 5. Conclusions

Due to the sparse distribution of ICESat data, it is difficult and even impossible to find individual conjugate points between ICESat data and optical images, an alternative simplified registration method based on point-to-line discrepancy correction in image space is presented for the registration of the two types of datasets. Compared with the indirect way of inconsistency elimination by orientation parameter correction through photogrammetric bundle adjustment, the direct discrepancy correction method is simpler for comprehension and easier for computation. There are also two schemes for direct discrepancy correction according to the space in which it is implemented, among which discrepancy correction in image space is simpler and more generic than that in object space and can work for individual images, whether they are stereo or mono. Moreover, it is suggested that the correction result be incorporated into imaging model via RPCs generation/regeneration for the convenience of applications.

The experimental results of using the ASTER images and ZY-3 images for registration with ICESat data show that sub-pixel level accuracies were achieved after registration. By comparing the DEMs derived from the stereo optical images and the ICESat data through elevation profile analysis along ICESat tracks, the results show a significantly higher consistency by using the regenerated RPCs with the correction model being incorporated after registration than using the original imaging models.

At the current stage, the matching features were extracted interactively. In further research, more effort could be paid to the automation of feature extraction and correspondences matching to improve the efficiency for practical applications.

## Figures and Tables

**Table 1:** Average distances (distance is the absolute residual in Equation (2)) between feature lines and projected feature points before and after registration using different transformation models (unit: pixel).

Transformation Model	ASTER NAD		ASTER BWD		ZY-3 NAD		ZY-3 FWD	
	Ctrl (9)	Chk (8)	Ctrl	Chk (8)	Ctrl (10)	Chk (8)	Ctrl (10)	Chk (8)
Before registration	4.2	5.6	4.2	5.2	1.5	1.9	0.7	1.0
Translation	2.3	3.8	1.9	2.3	1.1	1.6	0.6	0.9
Translation and scales	1.7	3.0	1.3	1.5	0.7	1.2	0.5	0.7
Similarity transformation	2.1	3.8	1.8	2.4	0.7	0.7	0.5	0.6
Affine transformation	0.5	0.8	0.6	0.8	0.7	0.6	0.3	0.5

**Table 2:** Elevation discrepancies before and after registration (unit: m).

		Before Registration	After Registration
ASTER vs. ICESat	Mean	−61.4	5.9
	Standard deviation	10.0	5.1
ZY-3 vs. ICESat	Mean	−28.3	−1.4
	Standard deviation	4.9	3.3
